# Evaluation of metastatic lymph nodes in cN0 thoracic esophageal cancer patients with inconsistent pathological lymph node diagnosis

**DOI:** 10.1186/s12957-020-01880-1

**Published:** 2020-05-29

**Authors:** Akiyuki Wakita, Satoru Motoyama, Yusuke Sato, Yuta Kawakita, Yushi Nagaki, Kaori Terata, Kazuhiro Imai, Yoshihiro Minamiya

**Affiliations:** Department of Thoracic Surgery, Akita University Graduate School of Medicine, 1-1-1 Hondo, Akita, 010-8543 Japan

**Keywords:** False-negative lymph node, Lymph node metastasis, Esophageal cancer

## Abstract

**Background:**

Preoperative clinical diagnosis of lymph node (LN) metastasis and subsequent pathological diagnosis are often not in agreement. Detection of false-negative LNs is essential in selecting an optimal treatment strategy, and most importantly, the presence of false-negative LN is itself a significant prognostic indicator. Therefore, at present, there is an urgent need to establish more accurate and individualized evaluation methods for LN metastasis.

**Methods:**

Of 213 cN0 patients who underwent curative esophagectomy without preoperative neoadjuvant treatment, 60 (28%) had LN metastasis diagnosed pathologically. There were 129 false-negative LNs, of which 85 were detectable by preoperative computed tomography (CT). We retrospectively investigated the distribution, frequency, and characteristics of pathologically positive nodes in patients with clinically N0 esophageal cancer.

**Results:**

The paracardial region was the most frequent region of false-negative LNs, accounting for 26% (22 LNs) of the total incidence. False-negative LNs distributed widely from the neck to the abdomen in patients with a primary tumor in the middle thoracic esophagus. In patients with a primary tumor in the lower thoracic esophagus, four false-negative LNs were detected in the superior mediastinum. When the short-axis diameter, shape, and attenuation patterns of the LNs were used as criteria for metastasis diagnosis, they were insufficient for an accurate diagnosis. However, false-negative LNs in the most frequently occurring sites are characterized by smaller short-axis, suggesting that accurate diagnosis cannot be made unless the diagnostic criteria for the short-axis are reduced in addition to shape and attenuation.

**Conclusions:**

Although restrictive to the most frequent regions of false-negative LNs occur, reducing size criterion and consideration of their shape and attenuation may contribute to improved diagnosis.

## Background

Regardless of advances in the comprehensive treatment of esophageal cancer, overall survival and preoperative 5-year survival remain poor [1, 2]. Lymph node stage is a key independent prognostic indicator in esophageal cancer. Beyond that, both the number and specific distribution of LN metastasis have been considered significant prognostic factors [3, 4]. For superficial esophageal cancer without LN metastasis, endoscopic mucosal resection (EMR) or endoscopic submucosal dissection (ESD) has shown results comparable to esophagectomy [5, 6]. In cases of locally advanced esophageal cancer without distant metastasis, the surgical approach and potential application of neoadjuvant therapy are dependent on the preoperative evaluation. Consequently, an accurate evaluation to identify those patients most likely to benefit from surgical intervention and those that are sufficient with minimally invasive treatment preserving esophagus is essential.

The presence of false-negative LNs hinders the actual cancer status and selection of an appropriate treatment. Despite its importance, preoperative clinical LN diagnosis and final pathological evaluation are often inconsistent [7]. In an earlier study, the rate of false-negative LN metastasis was reported to be 11–56% [8]. Clearly, a more accurate and individualized method or new criteria for conventional radiologic imaging are urgently required. To address this issue, we investigated the distribution, frequency, and characteristics of pathologically positive LNs in patients with clinically N0 esophageal cancer.

## Methods

### Patients

Between 2003 and 2016, 613 consecutive patients with thoracic esophageal cancer received esophagectomy at Akita University Hospital. Of those, 213 patients were diagnosed with cN0 thoracic esophageal cancer and received esophagectomy with extended 3-field lymphadenectomy without preoperative neoadjuvant therapy. Pathological diagnosis of 60 cN0 thoracic esophageal cancer patients showed positive LN metastasis. We enrolled those 60 patients in this study and retrospectively evaluated pathologically metastatic LNs and the patients’ clinical outcomes. Exclusion criteria were patients clinically diagnosed with LN metastasis and patients who received preoperative neoadjuvant chemotherapy or chemoradiotherapy. Informed consent for use of CT data for analysis was obtained from all patients included in the present study.

### Computed tomography scanning procedure

During the study period, we used two types of CT scanners: Discovery CT750HD or Discovery CT750HDA (GE Healthcare Japan, Tokyo, Japan). All patients were scanned from the neck to the pelvis. CT images were obtained using the helical technique with patients in a supine position. Slices with thicknesses of 1.25 mm (thin slice) and 5 mm were obtained before and after intravenous injection of 100 ml of non-ionic, iodinated contrast medium at 300–350 mg/ml. For contrast administration, we used an automatic injector that was set to deliver the contrast medium for 60 s. Scanning was begun at 95 s.

### Clinical diagnosis of LN metastasis in CT

Traditionally, LNs with short diameters **≥** 10 mm are considered to have cancer infiltration [9–11]. However, given the consistent frequency of metastatic nodes with short axes smaller than 10 mm, we reasoned that lowering the size criteria may contribute to more accurate diagnosis. In addition, metastatic LNs reportedly tend to be round [12], so combining axial ratio and LN size would be expected to increase sensitivity [13]. In the present study, therefore, LNs were diagnosed as metastatic when the short axis diameter was **≥** 8 mm, the shape of the node was round, and the internal density was heterogeneous. A preoperative diagnosis of LN metastasis was made by a multidisciplinary tumor board composed of gastroenterologists, surgeons, radiologists, oncologists, and pharmacists. The classification of regional LN station was made according to the Japanese Guidelines for Clinical and Pathologic Studies on Carcinoma of the Esophagus [14, 15], as presented in Table 1. Clinical TNM stage was determined according to the international Union against Cancer tumor-node-metastasis (TMN) Classification of Malignant Tumors (seventh edition) [16].

**Table 1:** Terminology used for lymph nodes in esophageal cancer

LN station No.	Location
Neck	
No. 100	Superficial lymph nodes of the neck
No. 101	Cervical paraesophageal lymph nodes
No. 102	Deep cervical lymph nodes
No. 103	Peripharyngeal lymph nodes
No. 104	Supraclavicular lymph nodes
Mediastinum	
No. 105	Upper thoracic paraesophageal lymph nodes
No. 106rec	Recurrent nerve lymph nodes
No. 106pre	Pretracheal lymph nodes
No. 106tb	Tracheobronchial lymph nodes
No. 107	Subcarinal lymph nodes
No. 108	Middle thoracic paraesophageal lymph nodes
No. 109	Main bronchus lymph nodes
No. 110	Lower thoracic paraesophageal lymph nodes
No. 111	Supradiaphragmatic lymph nodes
No. 112aoA	Anterior thoracic paraaortic lymph nodes
No. 112aoP	Posterior thoracic paraaortic lymph nodes
No. 112pul	Pulmonary ligament lymph nodes
Abdomen	
No. 1	Right paracardial lymph nodes
No. 2	Left paracardial lymph nodes
No. 3	Lesser curvature lymph nodes
No. 4sa	Lymph nodes along the short gastric vessels
No. 4sb	Lymph nodes along the left gastroepiploic artery
No. 4d	Lymph nodes along the right gastroepiploic artery
No. 5	Suprapyloric lymph nodes
No. 6	Infrapyloric lymph nodes
No. 7	Lymph nodes along the left gastric artery
No. 8a	Lymph nodes along the common hepatic artery (anterosuperior group)
No. 8p	Lymph nodes along the common hepatic artery (posterior group)
No. 9	Lymph nodes along the celiac artery
No. 10	Lymph nodes at the splenic hilum
No. 11p	Lymph nodes along the proximal splenic artery
No. 11d	Lymph nodes along the distal splenic artery
No. 12	Lymph nodes in the hepatoduodenal ligament

The left (L) and right (R) sides are considered separately for Nos. 101, 102, 104, 106rec, and 112pul

### Pathological examination for LN metastasis

For pathological examination, each LN was fixed in 20% buffered formalin, embedded in paraffin, sectioned, and stained with hematoxylin and eosin. Further investigation with immunohistochemical staining was performed for LNs that raised suspicion of cancer involvement. All dissected LNs were microscopically analyzed for metastatic disease by pathologists. Histopathological findings were classified according to the UICC TNM classification [16].

### Evaluation of LNs in this study

For each false-negative LN diagnosed microscopically, one-to-one correlation was made retrospectively with preoperative CT images. Lymph nodes not visualized by preoperative CT were excluded from the evaluation because of inconsistency with CT-pathological correlations. Lymph node size, configuration, and CT attenuation were measured to define the features of the false-negative LNs. We also evaluated the presence of the CT angiogram sign within the lesion in both the 1.25-mm thin slice and 5-mm slice axial images. To determine the size and configuration of the LNs, the long and short axes were measured, and the long-to-short axis ratios were calculated. The CT attenuation values of the nodes were compared with that of muscle attenuation and classified as hyper-, iso-, or hypo-attenuating.

### Surgery

Our standard operative procedure is right transthoracic or thoracoscopic esophagectomy with extended 3-field lymphadenectomy (bilateral neck including supraclavicular LN and mediastinal and abdominal lymph nodes). We commonly perform reconstruction by inserting a gastric tube via the posterior mediastinal or the retrosternal route.

### Statistical analysis

Continuous variables are presented as medians (minimum-maximum). Categorized data were analyzed using the Pearson’s Chi square test or Fisher’s exact probability test. All statistical analysis was performed using JMP 11 (SAS Institute Inc., Cary, NC, USA) and yielded two-sided *p* values. Values of *p* < 0.05 were considered statistically significant.

## Results

### Patient characteristics

The patients’ clinicopathological characteristics are summarized in Table 2. Of 213 cN0 patients who underwent curative esophagectomy without neoadjuvant treatment, 60 (28%) had total of 129 pathologically diagnosed metastatic LNs. That patient population included 51 (85%) males and 9 (15%) females with a median age of 67 years (range, 51–85 years). Four (7%) of the tumors were in the upper thoracic esophagus, 33 (55%) were in the middle thoracic segment, and 23 (38%) were in the lower thoracic segment. The median tumor size was 40 mm (range, 10–95 mm). The depth of the tumor invasion was pT1 in 29 (48%) patients, T2 in 7 (12%) patients, T3 in 22 (37%) patients, and T4 in 2 (3%) patients. Histological findings showed that in 49 (82%) patients the tumor was a squamous cell carcinoma, while in 5 (8%) patients it was an adenocarcinoma.

**Table 2:** Clinicopathological features of cN0 esophageal cancer patients

All patients (*n* = 213)	Lymph node involvement		*p*
	Positive (*n* = 60)	Negative (*n* = 153)	
Gender			0.712
Female	9	20	
Male	51	133	
Age at surgery	67 (51–85)	66 (39–81)	0.890
Tumor location			0.338
Upper	4	18	
Middle	33	79	
Lower	23	56	
Tumor size (mm)	40 (10–95)	35 (10–106)	0.146
Tumor depth (pT)			< 0.0001*
T1	29	123	
T2	7	10	
T3	22	18	
T4a	2	0	
T4b	0	2	
Lymph node metastasis (pN)			< 0.0001*
N0	0	152	
N1	45	0	
N2	12	1	
N3	3	0	
Pathological stage			< 0.0001*
IA	1	109	
IB	0	23	
IIA	0	18	
IIB	27	0	
IIIA	23	1	
IIIB	4	0	
IIIC	5	2	
Tumor histology			0.032*
Squamous cell carcinoma	49	141	
Adenocarcinoma	5	6	
Other	6	6	
Tumor differentiation of SCC			
G1	11	17	0.005*
G2	23	101	
G3	15	21	
N/A	0	2	
Prognosis			
Alive	40	124	0.017*
Deceased from esophageal ca.	10	7	
Deceased from other ca.	0	4	
Deceased from other diseases	10	18	

*Statistically significant

### Frequency and most likely locations of false-negative LNs

**Fig. 1:**
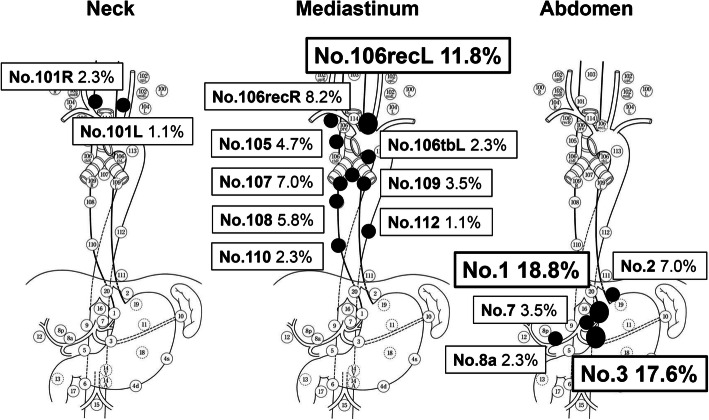
Distribution of false-negative LNs visualized on CT. Among the 85 false-negatives, the right paracardial LNs were most frequently metastatic (18.8%, 16/85LNs), followed by lesser curvature LNs (17.6%, 15/85LNs) and left recurrent nerve LNs (11.8%, 10/85LNs)

At total of 129 false-negative LNs were detected in 60 patients. Retrospectively examined, 85 (66%) of the LNs were visualized in the preoperative enhanced CT. Their distribution is summarized in Fig. 1. Sixteen (18.8%) false-negative LNs were detected in the right paracardial region. This was followed in descending order by the lesser curvature LNs (15/85, 17.6%), left recurrent nerve LNs (10/85, 11.8%), and right recurrent nerve LNs (7/85, 8.2%).

### Locations of false-negative LNs and the primary tumors

**Fig. 2:**
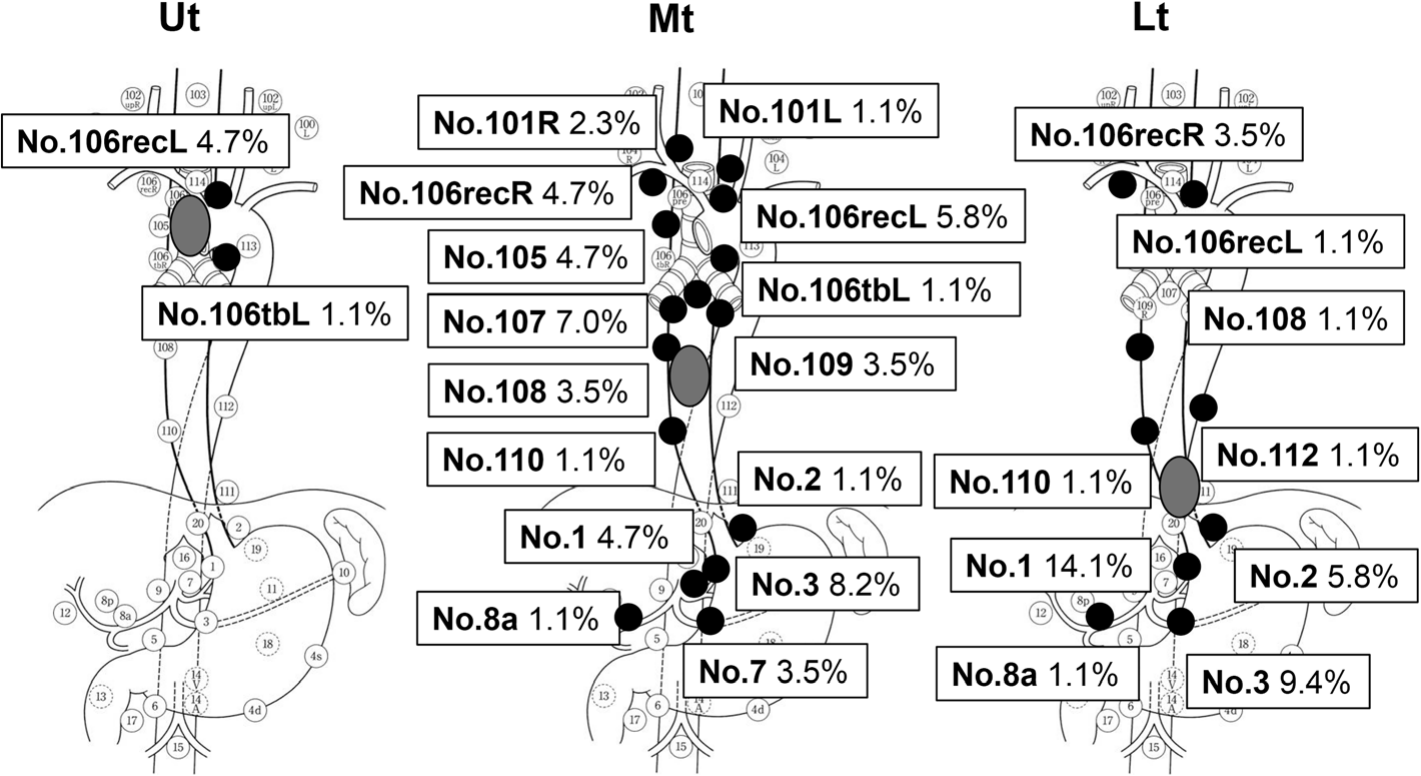
Distributions of false-negative LNs and location of the primary tumors. False-negative LNs associated with upper thoracic tumors were found in the superior mediastinal region. By contrast, with middle and lower thoracic tumors, false-negative LNs were detected in all three fields

The regions of false-negative LNs in patients with primary tumors in the upper, middle, or lower thoracic esophagus are summarized in Fig. 2. In patients with upper thoracic esophageal cancer, all five false-negative LNs were located within superior mediastinal region, four were left recurrent LNs, and 1 was a left tracheobronchial LN. In patients with a primary tumor in the middle thoracic esophagus, 46 false-negative LNs were widely distributed from the neck to the abdomen. In patients with a primary tumor in the lower thoracic esophagus, four of 34 false-negative LNs were detected in the superior mediastinal region. Three were right recurrent nerve LNs, and one was a left recurrent nerve LN.

### Size, shape, and attenuation values of false-negative LNs

**Fig. 3:**
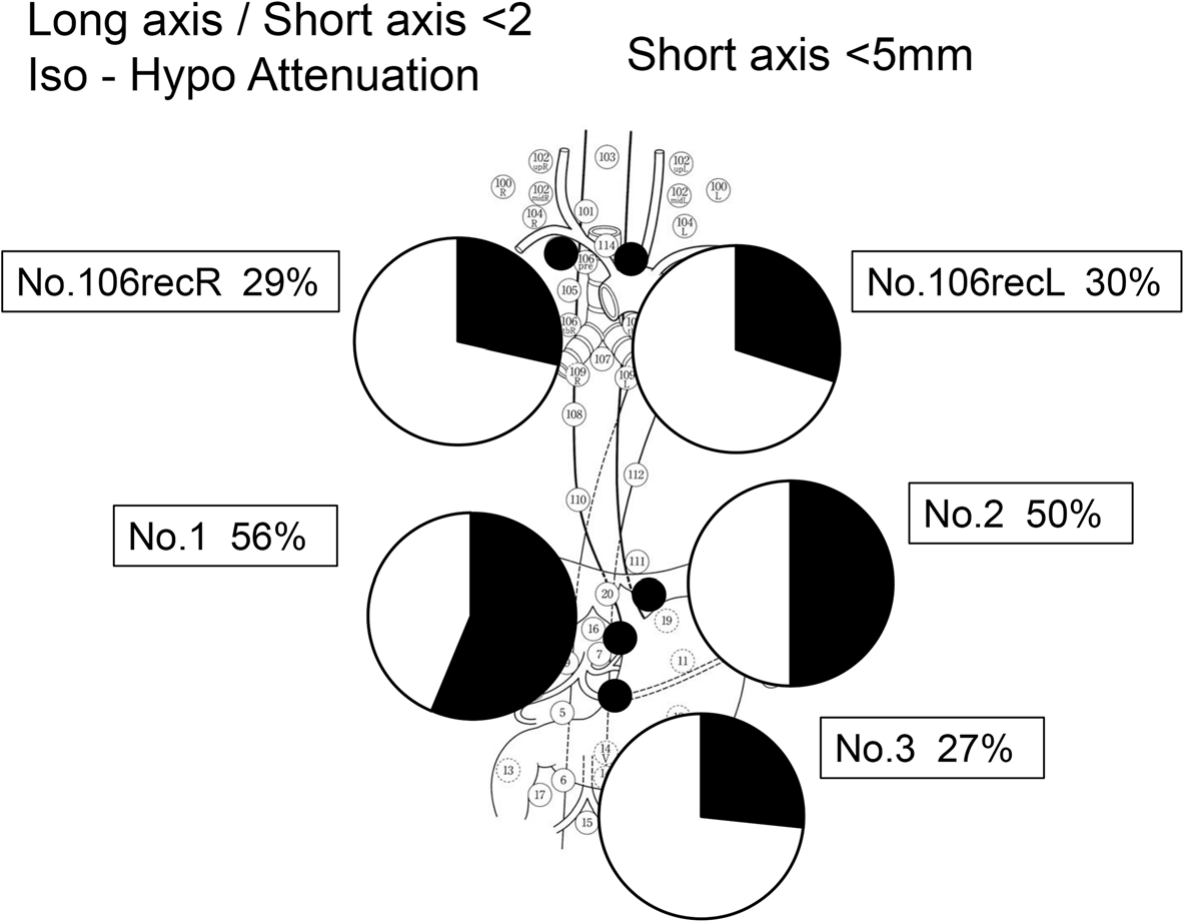
Focusing on the paracardial region, where LN metastasis most frequently occurred, 50–56% of false-negative LNs were small (< 5 mm), oval, and iso-hypo attenuated. This highlights the difficulty of diagnosing LN metastasis in this region

**Fig. 4:**
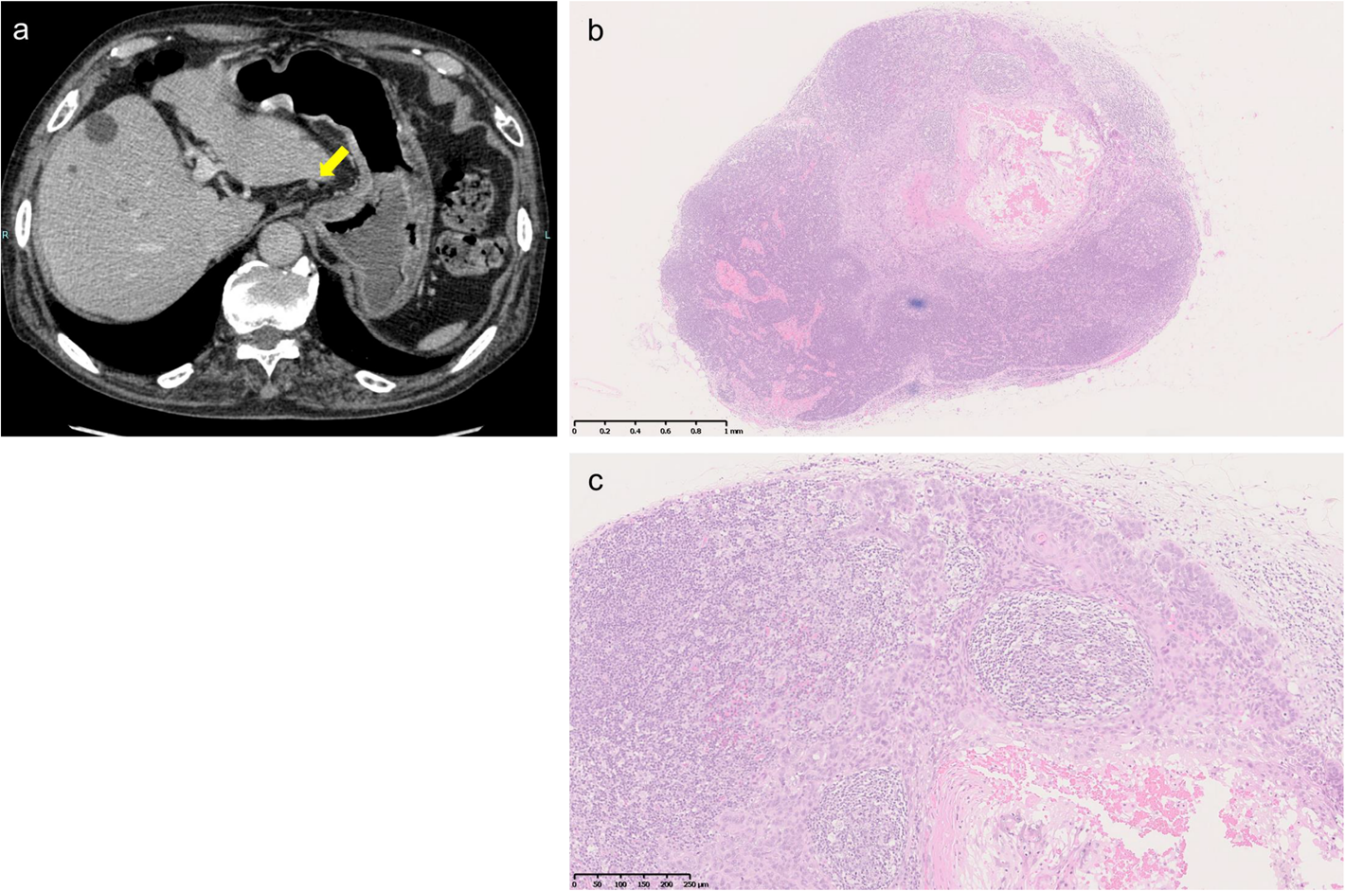
Representative CT findings and microscopic findings of false-negative LN. **a** CT indicates iso-attenuated oval LN in paracardial region (yellow arrow). **b** The size of the LN measured 3.5 mm in low-magnification image. **c** High-magnification image shows the area of metastasis within the false-negative LN

Features of false-negative LNs evaluated by CT are shown in Table 3. Understandably, 76 (89%) of the false-negative LNs did not exceed 8 mm in size. The long-to-short axis ratio was less than 2 in 73 (86%) of the 85 LNs. Fifty nodes (59%) were iso-attenuated, and 16 (19%) were hypo-attenuated. With addition of shape (round) and attenuation (iso-hypo) to the criteria for false-negative LNs, still only 5 (6%) of the 85 false-negative LNs would be regarded as metastatic. Particularly, 50–56% of false-negative LNs in the paracardial region had short diameters less than 5 mm and long-to-short axis ratios of less than 2, and they were iso-hypo-attenuated (Figs. 3 and 4). Upon further addition of a reduced CT size criterion (**≥** 5 mm) for false-negative LNs in the most frequent locations, 16 (30%) of the 54 false-negative LNs would be regarded as metastatic.

**Table 3:** Features of false-negative lymph nodes evaluated by CT

Positive for LN involvement (*n* = 85)			
	Neck (*n* = 3)	Mediastinum (*n* = 40)	Abdomen (*n* = 42)
Shape of LN			
Sphere	1 (1.2%)	9 (10.6%)	20 (23.5%)
Oval	2 (2.4%)	23 (27.1%)	18 (21.2%)
Flat	0	8 (9.4%)	4 (4.7%)
Size (short axis) of LN (mm)			
**≥** 10	0	1 (1.2%)	1 (1.2%)
< 10	3 (3.5%)	39 (45.9%)	41 (48.2%)
**≥** 8	0	2 (2.4%)	7 (8.2%)
< 8	3 (3.5%)	38 (44.7%)	35 (41.2%)
**≥** 5	2 (2.4%)	19 (22.3%)	21 (24.7%)
< 5	1 (1.2%)	21 (24.7%)	21 (24.7%)
Attenuation			
Hypo-	0	8 (9.4%)	8 (9.4%)
Iso-	3 (3.5%)	22 (25.9%))	25 (29.4%)
Hyper-	0	10 (11.8%)	9 (10.6%)

## Discussion

Our present study demonstrates that 28% of patients who underwent esophagectomy with LN dissection without neoadjuvant treatment had LNs falsely diagnosed as negative for metastasis, which is consistent with an earlier study [8]. False-negative LNs were most recognized in paracardial (Nos.1, 2) and paratracheal (No.106) region. In addition, it was shown that false-negative LNs in the most frequent region were smaller than those in other regions.

Due to the complex development of the lymphatic network around the esophagus, LN metastasis of thoracic esophageal cancer widely distributes from the neck to the abdomen [17, 18]. Indeed, our study showed that false-negative LNs were not limited to the vicinity of the primary tumor. Our assessments of nodal metastasis, taking into consideration the location of primary tumor, showed that false-negative LNs associated with middle thoracic esophageal tumors distributed broadly from the neck to abdomen. Moreover, in patients with a primary tumor in the lower thoracic esophagus, false-negative LNs were detected even in the superior mediastinum. Among the various regions for possible LN metastasis, false negatives were most frequently detected among the paracardial (Nos.1, 2) and paratracheal (No.106) LNs (26% and 22%, respectively). Focusing on the most frequent region (paracardial; Nos.1, 2), 50–56% of false-negative LNs were small (< 5 mm). This suggests that in the frequent regions of false-negative LNs, even small LNs may be metastatic when they are roundish and iso-hypo-attenuated. Because LN metastases are often microscopic, the cancer-involved nodes may be small, as in these cases. Kajiyama et al. reported that 37.2% of metastatic LNs were < 5 mm in diameter [19]. This makes diagnosis difficult at the preoperative stage.

Modalities such as endoscopic ultrasound (EUS), CT, and FDG-positron emission tomography (FDG-PET) are frequently used for LN diagnosis, and previous studies report that their diagnostic performance is not satisfactory. The results of meta-analyses have shown that EUS improves the sensitivity of LN diagnosis of metastasis from 84.7 to 96.7% when combined with fine needle aspiration (FNA) [20–22] but is limited to evaluation of LNs in the proximity of the esophageal or gastric wall. FDG-PET has the disadvantage that LNs adjacent to the tumor are difficult to discriminate from the tumor itself because of poor spatial resolution. Furthermore, FGD-PET is not suitable for diagnosis of small lymph node metastasis. FDG-PET has been reported to be detectable in tumors with a diameter **≥** 6 mm or tumors measuring **≥** 33mm^2^ [23, 24], making it difficult to diagnose small metastatic LNs.

CT scanning is the noninvasive and most common modality used to evaluate metastatic infiltration of LNs in esophageal cancer. Although detection of LN metastasis on CT depends primarily on the size criteria, past reports have shown that the sensitivity of CT is unsatisfactory when LNs greater than 10 mm are considered positive for metastasis [23, 25, 26]. Short axis diameter alone is insufficient for detection of normally sized metastatic LNs, nor can it distinguish between reactive hyperplasia and metastatic enlargement [27]. We therefore included the shape and attenuation patterns of the nodes to verify the presence of LN metastasis. The results show that using these parameters improves detection of metastatic LNs within areas where the frequency of false-negative LNs is comparatively high.

We assume that CT scanning will continue to play a dominant role with the introduction of artificial intelligence, when not only the size, but also clinical factors such as the distribution of false-negative LNs and their frequent regions will be considered for determination of LN metastasis. However, despite the technological advances made so far, diagnosis of LN metastasis using CT remains unsatisfactory [28], and the precise role of CT for assessing disease in a preoperative setting is still under discussion.

In order to diagnose metastatic LNs more accurately, further research and establishment of other methods that supplement the limitations of diagnostic imaging are also expected. We previously reported that the CRP 1846C>T genetic polymorphism is an independent factor associated with LN metastasis in thoracic esophageal squamous cell carcinoma. Patients carrying the CRP 1846 T/T genotype showed LN metastasis significantly more frequently than those with other genotypes. Moreover, the specificity was 91% for cN0 patients diagnosed using CT combined with CRP genetic polymorphism [29]. This combination may thus be a useful new approach to evaluating the risk of LN metastasis.

Our study has limitations including the nature of the retrospective study design. This study did not examine all cN0 patients who were pathologically positive for LN metastasis but limited them to those who did not receive preoperative neoadjuvant therapy. This could potentially affect accurately evaluating the characteristics of false-negative LNs.

## Conclusion

In summary, the accuracy of anatomical imaging for diagnosis of LN metastasis is yet not satisfactory. Careful consideration is therefore needed, especially for diagnosis of LNs in the paracardial (No.1, 2) and paratracheal (No.106) regions. Limited to the most frequent regions of false-negative lymph nodes occur, reducing size criterion and consideration of their shape and attenuation may result in more accurate lymph node diagnosis.

## Data Availability

The datasets used and/or analyzed during the current study are available from the corresponding author on reasonable request.
